# Persistent Oxygen Requirement beyond Prematurity: A Case of Acquired Pulmonary Vein Stenosis

**DOI:** 10.1155/2017/3106871

**Published:** 2017-03-14

**Authors:** Tyler A. Fick, Bernadette Richards, Carl H. Backes, Molly K. Ball

**Affiliations:** ^1^Department of Pediatrics, The Ohio State University Wexner Medical Center, Columbus, OH 43205, USA; ^2^The Heart Center at Nationwide Children's Hospital, Columbus, OH 43205, USA; ^3^Center for Perinatal Research, The Research Institute at Nationwide Children's Hospital, Columbus, OH 43205, USA

## Abstract

Acquired pulmonary vein stenosis is a rare cardiac defect and diagnosis can often be challenging, as many cases present with refractory or prolonged oxygen requirement over the expected course. Comorbid conditions can cloud this diagnosis further. Prognosis is poor for most patients. We present a case of idiopathic acquired pulmonary vein stenosis and discuss diagnostics, treatment options, and the need for further collaborative studies.

## 1. Background

Pulmonary vein stenosis (PVS) is a rare cardiac complication in which the intraluminal vessel diameter is narrowed in some or all of the pulmonary veins [1, 2]. This narrowing of the pulmonary veins impairs the return of oxygenated blood to the left atrium, resulting in pulmonary congestion, decreased oxygenation via increased venous pressure, and ultimately elevated pulmonary artery pressures. Cases of PVS have been reported as both congenital and acquired. Classically, acquired PVS has been observed in several specific populations. In adults, acquired PVS commonly results as a complication following radiofrequency ablation for arrhythmias, in cases of external compression of the pulmonary veins and in patients with sarcoidosis. In the pediatric population, diagnosis of PVS has largely been limited to comorbid congenital heart lesions or following surgical correction of anomalous pulmonary venous return [3].

However more recently, cases of acquired pulmonary vein stenosis without antecedent risk factors have been reported in published literature [4]. Several case reports document infants with normal pulmonary venous anatomy on original echocardiogram who go on to develop respiratory insufficiency and are subsequently found to have new-onset stenosis of some or all of the pulmonary veins [1, 3–6]. The exact etiology of acquired stenosis remains unknown, but some have theorized that increased blood flow states such as that from a high-volume patent ductus arteriosus (PDA) or ventricular septal defect (VSD), as well as inflammatory response cascades, represent critical aspects of PVS pathogenesis. Specifically, it has been suggested that vascular mediators such as vascular endothelial growth factor (VEGF) [3, 4, 7] lead to the intimal and medial fibromuscular proliferation seen on vascular pathology [5, 8, 9]. Prematurity and bronchopulmonary dysplasia (BPD) have also been implicated, but at current their specific roles in pathogenesis remain unclear [7, 10–14].

Universally, long-term prognosis for PVS is poor. Data suggests limited survival beyond 5 years [12, 13, 15, 16]. Furthermore, increasing number of pulmonary vessels involved, prematurity, and other comorbidities have each been shown to further decrease survival [7, 14, 17]. Both surgical correction and vascular stenting approaches have achieved limited success and are complicated by high rates of restenosis. Surgical marsupialization, gamma-interferon, and chemotherapy have additionally been tried with variable results. However, no therapeutic strategy or surgical intervention has proven successful in improving long-term prognosis or survival for patients with PVS [13, 15, 16, 18].

## 2. Case Presentation

 A 26-week and 0-day newborn male was born via cesarean section for nonreassuring fetal heart tones in the setting of severe maternal hypertension. Birth weight was 629 grams (<10th percentile based on Fenton preterm growth curve) consistent with severe intrauterine growth restriction (IUGR). Maternal serologies and substance screenings were unremarkable. A single dose of antenatal steroids was administered at least 6 hours prior to birth. The patient was intubated and surfactant administered upon delivery. His initial hospital course was complicated by grade IV intraventricular hemorrhage and Bell's Stage 1 necrotizing enterocolitis (NEC).

He was transitioned to continuous positive airway pressure (CPAP) by two weeks of life, required reintubation, and eventually was weaned back to CPAP respiratory support. However, he continued to require CPAP and supplemental oxygen through 36-week corrected gestational age (CGA), consistent with a diagnosis of bronchopulmonary dysplasia (BPD). At this time, an echocardiogram (ECHO) was obtained to evaluate for cardiac etiologies impeding oxygenation. Spectral Doppler and color flow echocardiogram showed a secundum atrial septal defect (ASD) as well as right atrial and right ventricular enlargement, consistent with pulmonary hypertension. Of note, normal pulmonary venous anatomy and connections were documented. Inhaled nitric oxide (iNO) and sildenafil were added to his therapeutic regimen.

**Figure 1:**
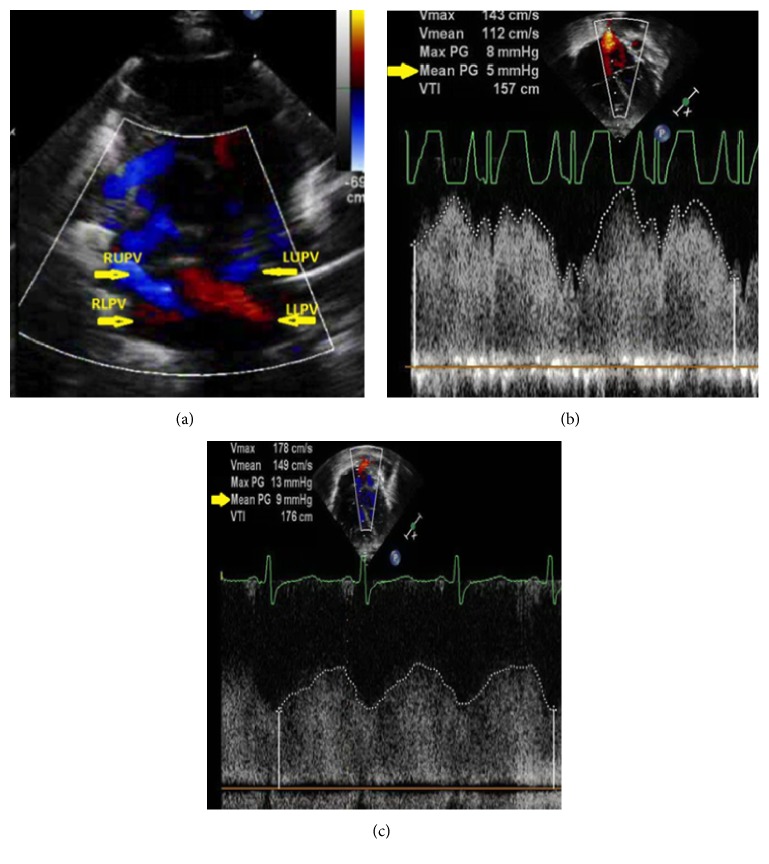
Transthoracic echocardiogram images demonstrating progressive pulmonary vein stenosis. (a) Color flow Doppler of pulmonary veins at initial diagnosis, with (b) measured mean pulmonary gradient (mean PG) of 5 mmHg and (c) increased mean pulmonary pressure gradient to 9 mmHg by 6 weeks after diagnosis.

Despite the addition of pulmonary vasodilator therapies, he continued to require positive pressure ventilation and supplemental oxygen support. With lack of clinical improvement, an echocardiogram was repeated at six weeks (42 weeks CGA) and diagnosed new left pulmonary vein stenosis. Subsequent computed tomography angiography and cardiac catheterization identified stenosis involving all five pulmonary veins, including a right middle pulmonary vein. This venous stenosis was felt to be the most likely cause of his ongoing oxygen and ventilatory requirements, outside of what would be his expected BPD clinical course. Serial echocardiograms over the next several months demonstrated progressive pulmonary vein stenosis (Figure 1).

Upon discussion with the family, the decision was made not to pursue catheterization or surgical treatment options in light of progressive stenosis and multivessel involvement with prematurity, BPD, and pulmonary hypertension comorbidities. Subsequent echocardiograms showed stabilization of stenosis. He was ultimately discharged to hospice home care with home CPAP at 11 months of age.

## 3. Discussion

PVS is a rare heart defect occurring in only 1.7 per 100,000 children under the age of two years [1, 2]. Acquired PVS comprises an even smaller subset, making it particularly unique [4]. In the pediatric population, acquired PVS patients often have a history of prolonged supplemental oxygen need with inability to wean or perceived lack of improvement along their expected clinical course. Typically, this persistent oxygen requirement prompts echocardiographic assessment. However, in cases of acquired PVS, initial echocardiogram may show normal pulmonary venous anatomy prior to the development of stenosis, making the diagnosis of PVS particularly difficult. Challenging the diagnosis further, NEC, BPD, and/or severe IUGR can be seen comorbid with PVS [14]. While the gold standard for diagnosis is cardiac catheterization, the risks of this procedure must be taken into consideration and may only be justified with a high index of suspicion, particularly given the frequency of cardiopulmonary morbidities in this population [19].

Tragically, PVS is widely considered a lethal diagnosis; the best-case scenario for 4-vessel involvement is sutureless surgical management, which portends a near 25% intraoperative mortality and only a 20% five-year survival [10]. Survival outcomes are markedly worse in patients with a diagnosis made in infancy, with more severe stenosis or increased vessel involvement or with existing comorbid conditions such as prematurity, NEC, or BPD [10–13, 20]. Treatment approaches have included surgical repair, chemotherapy, gamma-interferon therapy, and implantation of drug-eluting stents [2, 3, 5, 12, 17, 18, 21], but each has variable reported success and can be associated with significant complications. At current, no therapeutic or surgical intervention has clearly proven successful in improving long-term prognosis or survival. Heart-lung transplant as a curative therapy has recently come to light [22]; however many of these patients will die while waiting for a matched donor and at best will encounter the life-long risks associated with allogenic transplantation and immunosuppression.

Given the complexity of presentation and the possibility of multiple comorbid conditions, the diagnosis of PVS can be challenging for the medical team. Lack of effective longer-term therapeutic and surgical options with high morbidity and mortality make management decisions for the team and family equally difficult. For each patient, the complex decision regarding treatment and management recommendations involves weighing many risks, including the known lethality of acquired PVS with coexisting conditions, against those of potential interventions [15, 20]. Ultimately, for our patient, the family decided to pursue comfort care in lieu of the aforementioned treatment options because of the overwhelming suboptimal long-term prognoses. Little research exists to guide management decisions in an evidence-based manner; we feel that this represents one area in which advancements in the field can be readily achieved.

While PVS represents a rare pediatric diagnosis, there exists great need to look at multicenter collaborative data with appropriate subgrouping to better understand the natural history, influence of comorbidities, outcomes of therapeutic and surgical interventions, and short- and long-term complications in infants diagnosed with acquired PVS. Such data could not only improve diagnosis and prognostication but could provide important screening and surveillance recommendations [15, 23] as well as evidence-based recommendations for treatment. In knowing the scope, frequency, and timing of postrepair complications, we will be better able to develop plans for evaluation, monitoring, and prevention. For example, if systemic hypertension was identified as a risk factor for restenosis, strict blood pressure control postrepair may minimize cardiac and vascular remodeling.

Looking forward, collaborative data on pulmonary vein stenosis will provide a foundation to drive the development of clinical studies and therapeutic trials. While we acknowledge that the rarity of acquired PVS will make this task challenging, multicenter interdisciplinary collaboration is paramount to optimize study power and applicability of results. Given the severity of disease burden and limited efficacy of existing medical and surgical treatment options, great benefit can be achieved using collaborative data to aid both families and medical teams in optimizing the care and management of patients with pulmonary vein stenosis.

## Abbreviations

ASD: Atrial septal defect
BPD: Bronchopulmonary dysplasia
CGA: Corrected gestational age
CPAP: Continuous positive airway pressure
ECHO: Echocardiogram
iNO: Inhaled nitric oxide
PDA: Patent ductus arteriosus
PVS: Pulmonary vein stenosis
NEC: Necrotizing enterocolitis
VEGF: Vascular endothelial growth factor
VSD: Ventricular septal defect.

## Additional Points

*Table of Contents Summary*. The authors report acquired pulmonary vein stenosis in a preterm with bronchopulmonary dysplasia and pulmonary hypertension, highlighting diagnostic and therapeutic dilemmas and supporting multicentered data collection.
